# Global Comparative Phylogeography of *E. granulosus* s.s. Inferred From Mitochondrial DNA: Integrated Datasets of GenBank and Newly Characterized Isolates From Afghanistan

**DOI:** 10.1155/tbed/2401137

**Published:** 2026-07-16

**Authors:** Tharheer Oluwashola Amuda, Sayed Ajmal Qurishi, Guo-Dong Dai, Yao-Dong Wu, Wei-Gang Chen, Li-Qun Wang, Mughees Aizaz Alvi, Awab Ghulam Rahim, Li Le, Nian-Zhang Zhang, Wen-Hui Li, Bao-Quan Fu, Hong Yin, Wan-Zhong Jia, Xue-Nong Luo, Li Li, Hong-Bin Yan

**Affiliations:** ^1^ State Key Laboratory of Animal Disease Control and Prevention/College of Veterinary Medicine, Lanzhou University/Gansu Province Research Center for Basic Disciplines of Pathogen Biology/Key Laboratory of Veterinary Parasitology of Gansu Province/Key Laboratory of Veterinary Etiological Biology and Key Laboratory of Ruminant Disease Prevention and Control (West), Ministry of Agricultural and Rural Affairs/National Para-reference Laboratory for Animal Echinococcosis/Lanzhou Veterinary Research Institute, Chinese Academy of Agricultural Sciences, Lanzhou, 730046, China, caas.cn; ^2^ Department of Animal Nutrition and Production, Faculty of Agriculture, Afghan International Islamic University, Kabul, Afghanistan; ^3^ Department of Clinical Medicine and Surgery, University of Agriculture, Faisalabad, Pakistan, uaf.edu.pk; ^4^ Department of Public Health, Faculty of Medical, Afghan International Islamic University, Kabul, Afghanistan; ^5^ Mahidol-Oxford Tropical Diseases Research Unit Network (MORU-Tropical Network), Bangkok, Thailand; ^6^ Jiangsu Co-Innovation Center for Prevention and Control of Important Animal Infectious Disease, Yangzhou, 225009, China

**Keywords:** Afghanistan, cystic echinococcosis, *E. granulosus* s.s., livestock movement, mitochondrial DNA, phylogeography, transboundary transmission

## Abstract

Cystic echinococcosis (CE), caused by *Echinococcus granulosus* sensu stricto (*E. granulosus* s.s.), remains a major zoonotic disease of global public health importance. Afghanistan is considered endemic for CE; however, molecular data on circulating parasite populations are extremely scarce. This study aimed to characterize the genetic diversity, population structure, and global phylogeographic relationships of *E. granulosus* s.s. from Afghanistan within an integrated worldwide mitochondrial dataset. Hydatid cysts were collected from livestock hosts (cattle, sheep, and goats) in Afghanistan, and mitochondrial *cytochrome c oxidase subunit 1* (*cox1*) and *NADH dehydrogenase subunit 1* (*nad1*) genes were sequenced and analyzed alongside a comprehensive global dataset retrieved from GenBank. Genetic diversity indices, neutrality tests, median‐joining (MJ) haplotype (H) networks, sliding‐window analyses, and phylogenetic reconstructions based on concatenated mitochondrial sequences were employed. All Afghan isolates were identified as *E. granulosus* s.s., with a strong predominance of genotype G1 and only a single G3 isolate. Afghan populations exhibited moderate to high H diversity (Hd) but low nucleotide diversity, indicating closely related Hs separated by few mutations. Global H networks revealed star‐like topologies with extensive intercontinental H sharing and no host‐ or geography‐specific clustering. Phylogenetic and network analyses confirmed the genetic coherence of the G1 lineage worldwide and the limited diversity of G3. Sliding‐window analyses showed genome‐wide neutrality and absence of localized selective sweeps. Collectively, these findings position Afghan *E. granulosus* s.s. populations within a globally interconnected transmission network, highlighting the role of livestock movement and historical dispersal in shaping contemporary CE epidemiology. This study fills a critical regional knowledge gap and provides a robust phylogeographic framework to inform surveillance, control, and One Health interventions in endemic settings.

## 1. Introduction

Cystic echinococcosis (CE) is a neglected zoonotic disease caused by cestode parasites of the genus *Echinococcus* and remains a significant public health concern in both endemic and emerging regions worldwide. The disease is characterized by the development of hydatid cysts in the liver, lungs, and other organs of intermediate hosts, including livestock species (e.g., sheep, cattle, and goats) and humans, while canids, particularly domestic dogs serve as definitive hosts, perpetuating transmission through fecal shedding of infective eggs [1]. CE imposes substantial socioeconomic burdens due to livestock productivity losses, veterinary costs, and human morbidity [2]. It is particularly prevalent in pastoral and rural settings, where close human animal interactions, inadequate veterinary services, and informal slaughtering practices facilitate sustained transmission cycles [3, 4]. In addition, increasing livestock trade and transboundary movement of animals and dogs contribute to the dissemination and genetic mixing of parasite populations across endemic regions.

Within the *E. granulosus* sensu lato (s.l.) species complex, *Echinococcus granulosus* sensu stricto (*E. granulosus* s.s.) comprising genotypes G1 and G3 is recognized as the predominant causative agent of human CE globally [5–7]. These genotypes exhibit a broad host range and wide geographic distribution, infecting humans and a diversity of domestic and wild ungulates [8]. Despite their global importance, current molecular data remain geographically biased, with most studies concentrated in Europe, East Asia, and parts of the Middle East, while substantial gaps persist in Central and Western Asia [8, 9]. This uneven representation limits comprehensive understanding of global population structure and transmission dynamics.

Mitochondrial DNA markers, particularly the *cytochrome c oxidase subunit 1* (*cox1*) and *NADH dehydrogenase subunit 1* (*nad1*) genes, are widely employed in molecular epidemiology and phylogeographic studies of *E. granulosus* s.s. due to their high mutation rates, maternal inheritance, lack of recombination, and strong capacity to resolve intraspecific variation [10, 11]. These properties make them especially suitable for investigating genetic diversity, phylogenetic relationships, and patterns of gene flow across spatial scales. Accordingly, numerous studies have applied these markers to elucidate the global distribution and genetic structuring of *E. granulosus* s.s., revealing important insights into host connectivity and regional diversity [11–13]. Nevertheless, recent large‐scale analyses continue to highlight significant sampling gaps and emphasize the need to incorporate data from underrepresented endemic regions to improve phylogeographic inference [14].

Afghanistan represents a critical yet underexplored region in the molecular epidemiology of CE. Historical parasitological surveys have reported high prevalence of *E. granulosus* infection in livestock and stray dogs, particularly in Kabul during the 20th century; however, contemporary genetic data from human and animal hosts in Afghanistan are virtually absent from global sequence repositories. This gap is particularly important given that pastoral agriculture and smallholder livestock production key risk factors for CE transmission that constitute the economic backbone for a large proportion of the population, while unregulated movement of domestic animals and dogs remains common [15]. Furthermore, documented CE cases among Afghan residents, refugees, and international personnel highlight ongoing transmission and continued public health relevance [16, 17].

The absence of molecular data from Afghanistan limits the integration of regional parasite populations into global phylogeographic frameworks and constrains understanding of transboundary transmission dynamics and lineage connectivity. Given that genotype distribution and population structure of *E. granulosus* s.s. have important implications for parasite evolution, epidemiology, and control strategies, integrating data from poorly sampled endemic regions is essential for developing effective One Health interventions [18, 19].

Therefore, this study aims to investigate the global phylogeographic structure of *E. granulosus* s.s. using integrated mitochondrial (*cox1* and *nad1*) datasets and newly sequenced Afghan livestock isolates. By combining locally generated data with globally available sequences from NCBI, we seek to elucidate patterns of genetic diversity, population connectivity, and evolutionary relationships, thereby filling a critical geographic gap and providing evidence to support targeted control strategies for CE.

## 2. Materials and Methods

### 2.1. Sample Collection

**Figure 1:**
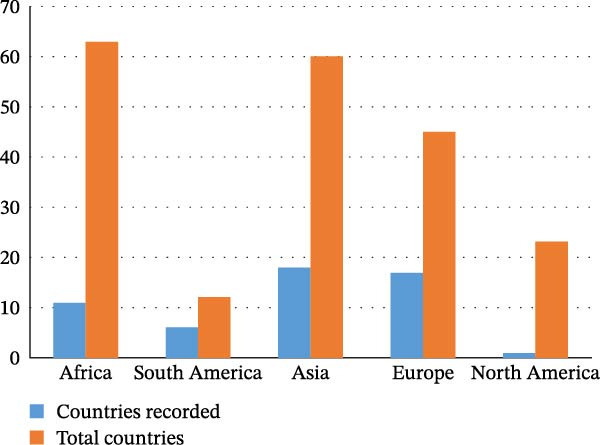
Continental geographical distribution of *E. granulossus* s.s. (G1 and G3) sequences submitted to NCBI.

Hydatid cyst samples were collected using a systematic random sampling approach from the livers and lungs of naturally infected livestock (cattle, sheep, and goats) during routine postmortem veterinary inspections at government‐administered abattoirs in Faryab Province, Northern Afghanistan (33.9391° N, 67.7100° E) (Figure 1). A total of 64 cysts were aseptically excised from individual animals (isolates information is available in Table 1). Protoscoleces and/or germinal layer tissues were applied onto Whatman FTA cards (GE Healthcare) following standard biosafety procedures. The cards were air‐dried at room temperature, individually packaged in sterile envelopes, and transported under ambient conditions to the Lanzhou Veterinary Research Institute (LVRI), Chinese Academy of Agricultural Sciences, China.

**Table 1:** Information on the isolates sampled from the Faryab province in Afghanistan.

Characteristics	Description	Frequency
Host	Cattle	22
	Goat	10
	Sheep	32
	Total	64
Location	Liver	23
	Lungs	30
	Liver/lungs	11
	Total	64
Sex	Male	29
	Female	35
	Total	64
Age	Young (1–2 years)	14
	Adult (3 years above)	50
	Total	64

### 2.2. DNA Extraction, Polymerase Chain Reaction (PCR) Amplification, and Sequencing

Genomic DNA was extracted from FTA cards by excising a small section (~2 mm^2^) containing parasite material and placing it into a sterile 1.5 mL microcentrifuge tube. DNA extraction was performed using the DNeasy Blood & Tissue Kit (Qiagen, Hilden, Germany) according to the manufacturer’s protocol, with minor adaptations for FTA card material. Extracted DNA was eluted in 50 μL of AE buffer and stored at −20°C until further analysis.

PCR amplification of partial mitochondrial *cox1* and *nad1* gene fragments was carried out in a final reaction volume of 25 μL containing 12.5 μL Premix Ex Taq Version 2.0 (Takara Bio, Kusatsu, Japan), 10 pmol of each forward and reverse primer, 1 μL of template DNA (~20–200 ng), and nuclease‐free water. Primer pairs were designed using Primer Premier 5 software based on the complete mitochondrial genome of *E. granulosus* s.s. (GenBank Accession: NC_044548) details in Table 2.

**Table 2:** Set of primers used for *Echinoccocus* spp. *nad*1 and *cox*1 amplification (GenBank ID NC_044548.1).

Primer ID	Primer sequence (5′‐3′)	Target gene	Product size (bp)	Mitochondrial region/position	GenBank ID	Reference
Echi‐*cox*1_F	AGTTACTGCTAATAATTTTGTGTCAT	*cox1*	1800	17,553 > 17,578	NC_044548.1	Present study
Echi‐*cox*1_R	ATGATGTAAAAGGCAAATAAACC			1716 < 1692		
Echi‐*nad*1_F	TAATGTTGATTATAGAAAATTTTCGTTTTACACGC	*nad1*	1200	15,818 > 15,852		
Echi‐*nad*1_R	CACAATTTATTATATCAAAGTAACCTGC			17,104 < 17,075		

PCR cycling conditions consisted of an initial denaturation at 95°C for 2 min, followed by 35 cycles of denaturation at 98°C for 15 s, annealing at 48°C for 30 s, and extension at 72°C for 90 s, with a final extension at 72°C for 10 min. Amplification products were separated by electrophoresis on 1.5% (w/v) agarose gels in 1 × TAE buffer, stained with GelRed (Biotium, USA), and visualized under ultraviolet illumination. Successful amplicons were purified and subjected to bidirectional Sanger sequencing by a commercial sequencing service (Beijing Tsingke Biotechnology Co., Ltd., China), following established protocols [20].

### 2.3. Sequence Assembly, Alignment, and Species Confirmation

Raw chromatograms were visually inspected, trimmed, and assembled into consensus sequences using DNASTAR v7.1 (Lasergene) and Unipro UGENE v1.29.0. Sequence quality was assessed to identify ambiguous base calls and sequencing artifacts. Multiple sequence alignments were performed using BioEdit v7.2.6, with manual adjustment where necessary [21, 22]. Species identity of each isolate was confirmed by querying the nucleotide sequences against the GenBank database using BLASTn (https://blast.ncbi.nlm.nih.gov/Blast.cgi), ensuring accurate assignment to *E. granulosus* s.s..

### 2.4. Retrieval and Curation of Global Mitochondrial Datasets

To place Afghan isolates within a global phylogeographic framework, mitochondrial *cox1* and *nad1* sequences of *E. granulosus* s.s. were retrieved from the NCBI GenBank database (last accessed: December 6, 2025). Searches employed keyword‐based queries (e.g., “*E. granulosus cox1*” and “*E. granulosus nad1*”) combined with organism filters and sequence length criteria.

Sequences were included if they (i) were assigned to *E. granulosus* s.s. (G1–G3 complex), (ii) contained geographic metadata at least at the country level, and (iii) exhibited acceptable sequence quality with minimal ambiguous nucleotides. Duplicates, laboratory strains lacking geographic information, and severely truncated sequences were excluded. Metadata including accession number, country, continent, host species, and year of submission were compiled for downstream analyses (Tables 3–6).

**Table 3:** Global distribution of *E. granulosus* s.s. isolates from Asia insights from NCBI.

Continent	Country	Year	Genotype (host)	Gene marker	GenBank ID
Asia	Nepal	2010	G1 (water buffalo)	*cox1*	AB551110
	Pakistan	2020	G1 (goat)	*cox1*	MW407999
	India	2009	G1 (goat)	*nad1*	GQ168806
	Iran	2022	G1 (human)	*nad1*	MZ927656
	Mongolia	2018	G1 (human)	*nad1*	MG672255
	Mongolia	2013	G1 (human)	*cox1*	AB893244
	Mongolia	2006	G1 (dog)	*cox1*	AB271235
	Australia	2018	G1 (dog) Dingo	*nad1*	MG672263
	Indian	2018	G1 (buffalo)	*nad1*	MG672260
	Kazakhstan	2018	G1 (human)	*nad1*	MG672257
	Iran	2018	G1 (goat/camel/buffalo)	*nad1//cox1*	MG672256
	Iraq	2012	G1 (sheep)	*cox1*	JX878689
	Australia	2019	G1 (sheep)	*cox1*	NC_044548
	Oman	2015	G1 (camel)	*nad1*	KR822179
	China	2013	G1 (human)	*cyto B*	KJ556990
	China	2013	G1 (human)	*nad5*	KC756236
	Afghanistan	2025	G1 (sheep)	*nad1*	PQ821704^a^
	Pakistan	2022	G1–G3 (human)	*cyto B*	OP580509
	Pakistan	2019	G1 (cattle)	*nad1*	MN886277
	Uzbekistan	2019	G1 (sheep)	*nad1*	MN696628
	China	2017	G1 (sheep)	*nad1*	MN269986
	China	2015	G1 (sheep)	*cox1*	KJ628374
	Russia	2006	G1(Cat/sheep)	*cox1*	AB622277
	Russia	2013	G1 (human)	*cox1*	AB777908
	India	2013	G1 (human)	*cox1*	KC422645
	China	2012	G1 (human)	*cox1*	AB688618
	Palestine	2012	G1 (sheep)	*cox1*	KC109659
	Iraq	2024	G1 (human)	*nad4*	PP391482
	Syria	2024	G1 (human)	*cox1*	PP385950
	Kazakhstan	2023	G1 (cattle)	*nad1*	OR294927
	China	2020	G1 (sheep)	*nad1*	OQ355550
	Lebanon	2021	G1 (sheep)	*cox1*	MW428247
	Palestine	2016	G1 (human)	*cox1*	KX522619

aFrom this study.

**Table 4:** Global distribution of *E. granulosus* s.s. isolates from Europe insights from NCBI.

Continent	Country	Year	Genotype (host)	Gene marker	GenBank ID
Europe	Italy	2022	G1 (sheep)	*nad5*	ON630437
	Greece	2018	G1 (sheep)	*nad1*	MG672282
	Poland	2017	G1 (sheep)	*cox1*	KJ831062
	Germany	2016	G1 (water vole)	*cox1*	JF747248
	Italy	2005	G1 (sheep)	*nad1*	DQ154008
	Portugal	2010	G1 (sheep)	*12S rRNA*	FR667944
	Turkey	2018	G1 (sheep)	*nad1*	MG672195
	Albania	2018	G1 (sheep)	*nad1/cox1*	MG672141
	Finland	2018	G1 (human)	*nad1/cox1*	MG672132/KY766884
	Romania	2018	G1 (cattle)	*cox1*	MG672131
	Greece	2018	G1 (sheep)	*cox1*	MG672130
	France	2017	G1 (cattle)	*cox1*	KY766889
	France	2024	G1 (human)	*cox1*	PP990341
	Iceland	2023	G1 (sheep)	*cox1/nad1*	OQ269593
	Italy	2023	G1 (sheep)	*cox1*	OR640381
	Turkey	2023	G1 (human)	*cox1*	OR126887
	Slovenia	2020	G1 (human)	*nad1*	MT239142
	Spain	2016	G1 (goat)	*cox1*	KU925422
	Moldova	2014	G1 (not stated)	*cox1*	KJ782437
	Poland	2015	G1 (human)	*nad1*	KT780300
	Greece	2025	G1 (human)	*nad1*	PV167800
	United Kingdom	2014	Dog	*cox1*	KP101619
	Spain	2018	G1 (goat)	*nad1*	MG672154
	Ukraine	2021	G1 (pig)	*nad1*	MW542593

**Table 5:** Global distribution of *E. granulosus* s.s. isolates from Africa insights from NCBI.

Continent	Country	Year	Genotype (host)	Gene marker	GenBank ID
Africa	Algeria	2017	G1 (human)	*nad1*	MG672292
	Algeria	2018	G1 (human)	*cox1*	MG672292
	Egypt	2014	G1 (sheep)	*nad1*	AB921125
	Tunisia	2015	G1 (cattle)	*nad1*	KT363807
	Tunisia	2018	G1 (human)	*cox1*	MG672275
	Morocco	2018	G1 (cattle)	*cox1*	MG672142
	Tanzania	2021	G1 (cattle)	*cox1*	MW729426
	Nigeria	2025	G1 (cattle)	*cox1*	PV208408
	Egypt	2024	G1 (human)	*nad1*	PP814720
	Kenya	2017	G1 (goat)	*cox1*	MH274965
	Egypt	2014	G1 (sheep)	*ACTII*	AB921051
	Mauritania	2005	G1 (human)	*ACTII*	DQ341551
	Tunisia	2016	G1 (dog/cattle)	*Nad1*	KU169240
	Ethiopia	2007	G1 (cattle)	*ACTII*	DQ341540
	Ethiopia	2011	G1 (sheep)	*cox1*	AB650529
	Tunisia	2021	G1 (human)	*Eg95-6*	MZ890124

**Table 6:** Global distribution of *E. granulosus* s.s. isolates from South America and North America insights from NCBI.

Continent	Country	Year	Genotype (host)	Gene marker	GenBank ID
South America	Argentina	2020	G1 (pig)	*cox1*	MT796074
	Mexico	2018	G1 (pig)	*nad1*	MG672259
	Argentina	2018	G1 (sheep)	*nad1*	MG672258
	Peru	2021	G1 (*Lama glama*)	*cox1*	MW732663
	Chile	2017	G1 (cattle)	*cox1*	KY766890
	Brazil	2015	G1 (cattle/pig)	*cox1*	KT382540
	Peru	2011	G1 (human)	*nad1*	JF946624
North America	USA	2023	G1 (cattle)	*nad5*	OR400700

### 2.5. Genetic Diversity and Neutrality Tests

Genetic diversity indices, including the number of haplotypes (Hs), H diversity (Hd), and nucleotide diversity (π), were calculated using DnaSP v6. Neutrality tests, including Tajima’s *D* and Fu’s *F*_s_ statistics, were employed to assess deviations from neutral evolution and infer historical demographic patterns [23]. Significance was evaluated based on 1000 coalescent simulations.

The median‐joining (MJ) H networks for *cox1*, *nad1*, and concatenated (*cox1–nad1*) sequences were constructed using PopART (http://popart.otago.ac.nz) to visualize genealogical relationships among Hs [20].

### 2.6. Phylogenetic Reconstruction and Evolutionary Analyses

Phylogenetic analyses were conducted using individual gene alignments (*cox1* and *nad1*) and concatenated datasets (*cox1–nad1*) for samples from Afghanistan while sequence data retrieved from NCBI were analyzed as individual gene. The most appropriate nucleotide substitution models were determined using Model Finder/Model Test based on Bayesian Information Criterion (BIC) in IQ‐TREE or MEGA X.

Maximum likelihood (ML) phylogenies were inferred using IQ‐TREE or RAxML with 1000 bootstrap replicates to assess node support. Bayesian phylogenetic inference was performed in MrBayes v3.2, employing two independent runs of four Markov chains over 5 million generations, sampling every 1000 generations. Convergence was assessed by examining effective sample sizes (ESS > 200), and the first 25% of samples were discarded as burn‐in.

Additionally, neighbor‐joining (NJ) trees were constructed in MEGA 11 using the method of Saitou and Nei [24], with bootstrap support assessed over 1000 replicates [25]. Phylogenetic trees were visualized and annotated using FigTree and iTOL.

## 3. Results

### 3.1. Sample Collection, Molecular Identification, and Continental Distribution of *E. granulosus* s.s.

A total of 64 hydatid cyst isolates were randomly collected from livestock hosts, including cattle (*n* = 22), sheep (*n* = 32), and goats (*n* = 10) within the study region (Table 1). PCR amplification of the mitochondrial *nad*1 and *cox*1 genes yielded amplicons of 894 bp and 1674 bp, respectively. Following sequencing and BLAST‐based identification, 62 isolates were successfully amplified and characterized, all of which were assigned to *E. granulosus* s.s.. Among these, 61 isolates belonged to genotype G1, whereas a single isolate was identified as genotype G3, indicating the strong predominance of the G1 lineage circulating in Faryab Province livestock populations and possibly by extension the rest of Afghanistan as a result free animal movement.

**Figure 2:**
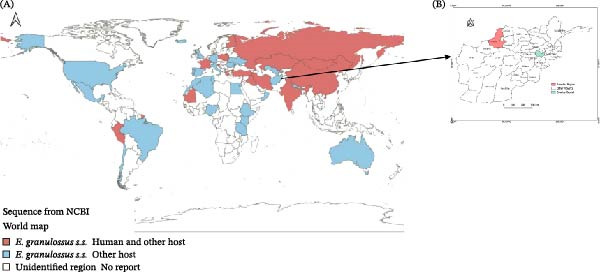
(A) Global distribution *E. granulosus* s.s. based on mitochondrial genome sequences: insights from integrated NCBI database. (B) Map of Afghanistan showing the region where sampling was conducted. The program QGIS 3.32.2 of QGIS was used to create the map.

The continental distribution of *E. granulosus* s.s. sequences retrieved from NCBI is summarized in Table 3 and illustrated in Figure 1. Asia and Europe exhibited the broadest representation, with sequence reported in NCBI from 20 and 16 countries, respectively. Africa followed with records from 10 countries, while South America showed reports from six countries. North America displayed the most restricted distribution, with reports from a single country. The global spatial distribution of sequences and the geographic origin of Afghan samples are shown in Figure 2, generated using QGIS v3.32.2.

### 3.2. Genetic Diversity and H Structure of Afghan *E. granulosus* Isolates

Complete mitochondrial sequences of *cox*1 (1674 bp), *nad*1 (894 bp), and concatenated *nad1–cox1* regions from the Afghan isolates were analyzed. A total of 13, 6, and 11 polymorphic sites were detected in the *cox1, nad1*, and concatenated datasets, respectively (Table 7). Parsimony‐informative sites accounted for 61.5% (8/13) in *cox*1, 16.7% (1/6) in *nad*1, and 54.5% (6/11) in the concatenated dataset.

**Table 7:** Diversity and neutrality indices for *E. granulosus* s.s. populations from Afghanistan.

Feature/index	*cox*1 (1674 bp)				*nad*1 (894 bp)				*cox*1–*nad*1 (2568 bp)			
	Cattle	Sheep	Goat	Total	Cattle	Sheep	Goat	Total	Cattle	Sheep	Goat	Total
Number of isolates	12	16	5	33	6	13	5	24	4	9	3	16
Number of mutations	9	12	1	13	1	3	4	6	6	10	6	11
Parismony informative sites	6	6	0	8	1	1	1	1	—	7	0	6
Number of haplotypes	4	6	2	9	2	3	3	5	2	4	3	6
Haplotype (gene) diversity (hd)	0.682	0.783	0.4	0.782	0.600	0.564	0.8	0.630	0.667	0.778	1	0.817
Variance of haplotype diversity (Var Hd)	0.01039	0.00521	0.05632	0.00255	0.01667	0.01248	0.02688	0.00426	0.04167	0.01209	0.07407	0.00326
Nucleotide diversity (π)	0.00207	0.00183	0.00024	0.00178	0.00067	0.00093	0.00201	0.00106	0.00157	0.00154	0.00157	0.00137
Tajima’s *D* (*p*‐value)	0.58407	−0.65109	−0.8165	−0.3011	1.4451	−0.47825	−0.41017	−1.2648	2.15629	0.26509	—	0.16333
Fu’s *F*_s_	2.358	0.41	0.09	−0.586	0.795	0.375	0.46900	−1.061	3.526	1.93200	0.134	0.813

**Figure 3:**
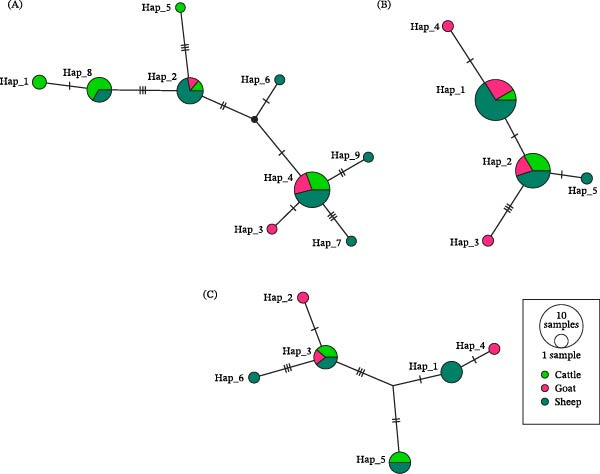
Median‐joining network of *E. granulosus* haplotypes from Afghan based on the *cox*1 (1674 bp) (A), *nad*1 (894 bp) (B), and *cox*1–*nad*1 (2568 bp) (C) mitochondrial genes. Circle sizes are proportional to the corresponding haplotype frequencies with hatch marks representing the number of mutations.

The number of Hs identified was nine for *cox*1, five for *nad*1, and six for the concatenated *nad*1–*cox*1 sequences. MJ network analysis revealed the presence of dominant central Hs accompanied by multiple low‐frequency variants. In the *cox*1 network, Afghan‐Hap4 was the most frequent H (13/33 sequences), while five Hs occurred as singletons. For *nad*1, Afghan‐Hap1 predominated (12/24 sequences), whereas three Hs were detected only once. In the concatenated dataset, Afghan‐Hap3 was the most frequent H (Figure 3). Representative H sequences were deposited in GenBank under accession numbers PQ305597–PQ305606 (*cox*1) and PQ821697–PQ821702 (*nad*1).

### 3.3. H and Nucleotide Diversity and Neutrality Tests

Hd values were moderate to high, with estimates of 0.782 (*cox*1), 0.630 (*nad*1), and 0.817 (concatenated *nad*1–*cox*1), while nucleotide diversity (π) values remained comparatively low (0.00255–0.00426), indicating the presence of multiple closely related Hs separated by few mutational steps.

Neutrality tests revealed negative but statistically nonsignificant Tajima’s *D* and Fu’s *F*_s_ values for the individual *nad*1 and *cox*1 loci (*p*  > 0.05), suggesting no strong deviation from neutrality at single‐gene resolution. In contrast, the concatenated *nad*1–*cox*1 dataset yielded slightly positive values, consistent with a genetically stable population under weak demographic or selective pressures.

### 3.4. Phylogenetic Analysis Based on Concatenated Mitochondrial Sequences

Phylogenetic relationships among Afghan isolates were inferred using concatenated full‐length *cox1–nad1* sequences together with reference sequences retrieved from GenBank. The analysis was performed in MEGA X, with *Taenia saginata* (PP391461) used as the outgroup.

**Figure 4:**
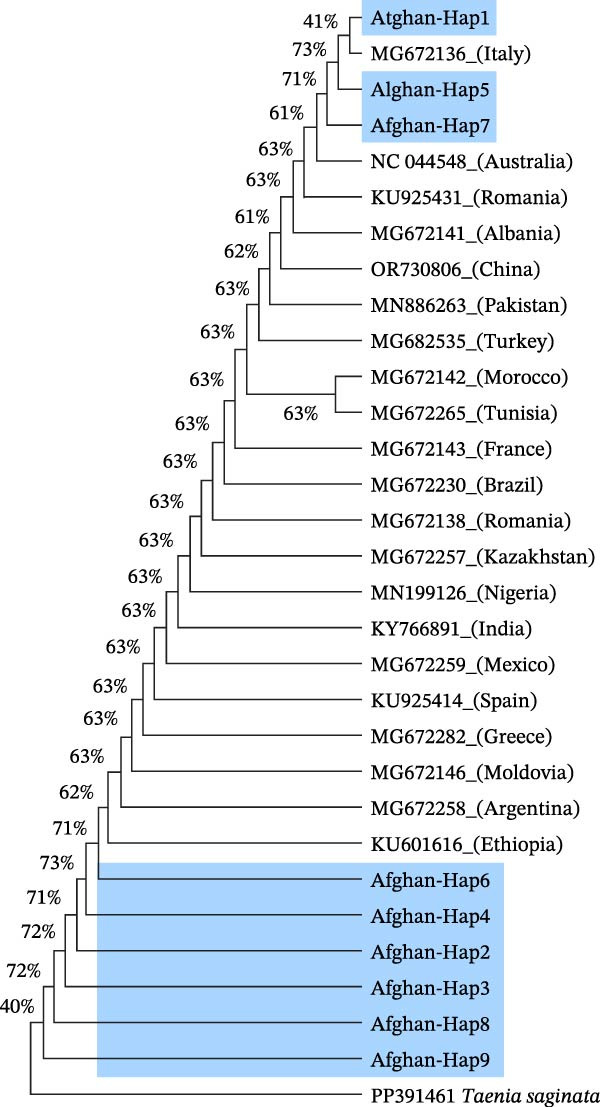
The phylogenetic relationships were inferred using MEGA‐X software based on concatenated full‐length nucleotide sequences (*cox*1–*nad*1), with *T. saginata* (PP391461) serving as the outgroup.

The resulting phylogenetic tree (Figure 4) clearly resolved *E. granulosus* s.s. into distinct genotype clusters, with all Afghan G1 isolates forming a well‐supported monophyletic clade alongside global G1 reference sequences. The single Afghan G3 isolate clustered separately with other G3 sequences, confirming its genotype assignment and genetic distinctness from the dominant G1 lineage. No host‐specific clustering was observed within the G1 clade, as isolates from sheep, cattle, and goats were interspersed throughout the tree.

### 3.5. Global H Networks, Genetic Diversity, and Phylogeographic Structure

#### 3.5.1. Genotype G3 (nad1): Global H Structure and Diversity

The MJ network constructed from global *nad1* sequences of *E. granulosus* genotype G3 revealed a compact and weakly structured topology, characterized by minimal mutational separation among Hs (Supporting Information 1: Figure S1). A total of 14 sequences representing four Hs were included in the analysis (Table 8). The Afghan isolate (PV610729) constituted a unique H, differing by one to two mutational steps from isolates originating from Asia, Europe, and Africa, with no evidence of geographic clustering.

**Table 8:** Genetic diversity indices for *E. granulosus* s.s. (G1 and G3 genotypes) based on mitochondrial *cox*1 (1876 bp) and *nad*1 (894 bp) sequences retrieved from global NCBI datasets.

Features	G1		G3
	*cox1*	*nad1*	*nad1*
Number of isolates	43	31	14
Number of mutations	13	107	3
Parismony informative sites	2	4	0
Number of haplotypes	6	10	4
Haplotype (gene) diversity (hd)	0.221	0.546	0.396
Variance of haplotype diversity (Var Hd)	0.0071	0.01166	0.02521
Nucleotide diversity (π)	0.02472	0.0333	0.00048
Tajima’s *D* (*p*‐value)	−2.36964	−2.78476	−1.67053
Fu’s *F*_s_	−2.348	2.036	−2.288

Consistent with the network topology, genetic diversity indices indicated limited variability within the G3 lineage. Only three mutations were detected across the *nad1* dataset, with no parsimony‐informative sites identified (Table 8). Hd was moderate (Hd = 0.396), whereas nucleotide diversity was extremely low (π = 0.00048), reflecting close genetic relatedness among global G3 isolates. Neutrality statistics showed negative Tajima’s *D* (−1.67053) and Fu’s *F*_s_ (−2.288) values.

#### 3.5.2. Sliding‐Window Analysis of Nucleotide Diversity and Neutrality Statistics

**Figure 5:**
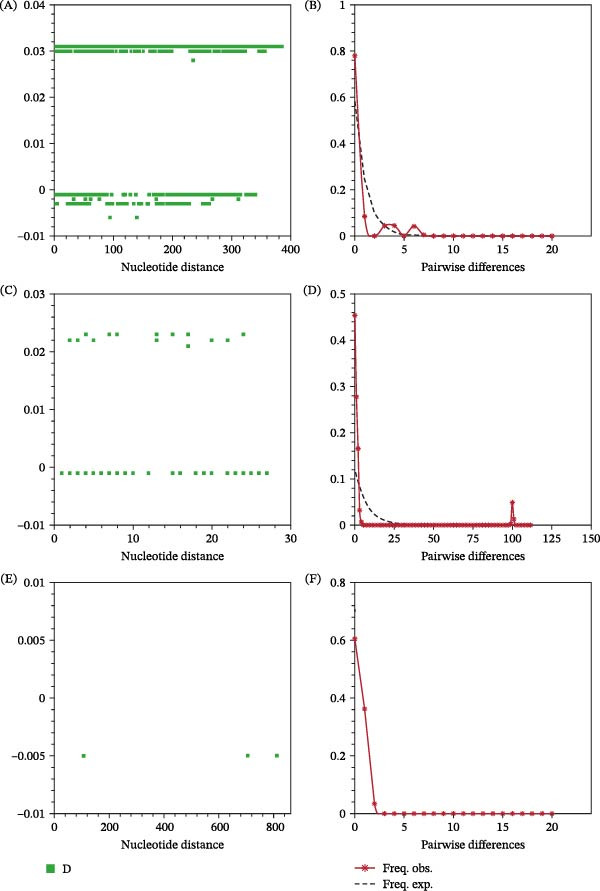
Comparative sliding‐window analysis of *nad1* and *cox1* genes in *E. granulosus* genotypes G1 and G3. (A, C, E) Tajima’s D and (B, D, F) nucleotide diversity (π) across the *nad1* (894 bp) and *cox1* (1876 bp) loci for G1 (A–D) and the *nad1* locus for G3 (E, F), based on publicly available NCBI sequences.

To further investigate fine‐scale patterns of genetic variation and neutrality across mitochondrial loci, sliding‐window analyses of Tajima’s *D* and nucleotide diversity (π) were performed for the *nad1* (894 bp) and *cox1* (1876 bp) genes in *E. granulosus* genotypes G1 and G3 using publicly available global sequences (Figure 5).

For G1 isolates, Tajima’s *D* values across both *nad1* (Figure 5A) and *cox1* (Figure 5C) regions were consistently close to zero or weakly negative, with no pronounced peaks or troughs, indicating genome‐wide neutrality across mitochondrial loci. Corresponding nucleotide diversity and mismatch distribution plots (Figure 5B,D) displayed unimodal to slightly right‐skewed distributions, characterized by a high frequency of low pairwise differences and a rapid decline toward higher divergence classes.

In contrast, the G3 *nad1* dataset exhibited markedly reduced nucleotide diversity across all sliding windows (Figure 5F), with Tajima’s *D* values fluctuating narrowly around zero (Figure 5E). The mismatch distribution for G3 was sharply unimodal, reflecting extremely low intragenotype divergence and limited accumulation of mutations.

Collectively, these patterns indicate genome‐wide homogeneity, absence of localized selective sweeps, and a recent shared evolutionary history, particularly pronounced in the G3 lineage.

#### 3.5.3. Global H Structure of *E. granulosus* s.s. (G1)

The MJ network constructed from global *nad*1 sequences of *E. granulosus* s.s. genotype G1 revealed a pronounced star‐like topology dominated by one or more central Hs shared across continents (Supporting Information 2: Figure S2). Numerous low‐frequency Hs radiated from these central nodes, each separated by few mutational steps, indicative of shallow genetic divergence.

Analysis of the *cox*1 gene provided higher haplotypic resolution while retaining the same overall network architecture (Supporting Information 3: Figure S3). Several high‐frequency *cox*1 Hs were shared across Asia, Europe, Africa, and the Americas, whereas most region‐specific Hs occurred at low frequencies and appeared as derived variants.

Geographic mapping of global *cox*1 Hs further confirmed extensive intercontinental H sharing, with Asia and Europe exhibiting the greatest H richness (Supporting Information 4: Figure S4). No clear continental segregation of Hs was observed, and identical Hs were frequently detected across distant geographic regions.

#### 3.5.4. Comparative Global Genetic Diversity of G1 and G3 Genotypes

Comparative analysis of genetic diversity indices based on mitochondrial *cox1* and *nad1* sequences highlighted contrasting patterns between G1 and G3 genotypes (Table 8). The G1 genotype exhibited greater overall variability, with 13 mutations and six Hs detected in the *cox*1 dataset (*n* = 43), and 107 mutations and 10 Hs in the *nad*1 dataset (*n* = 31). Hd was low to moderate (*cox1* Hd = 0.221; *nad1* Hd = 0.546), while nucleotide diversity values remained low (*cox1* π = 0.02472; *nad1* π = 0.0333).

Neutrality tests for G1 revealed significantly negative Tajima’s *D* values for both *cox*1 (−2.36964) and *nad*1 (−2.78476). Fu’s *F*_s_ values were negative for *cox*1 (−2.348) but positive for *nad*1 (2.036). In contrast, the G3 genotype displayed consistently low diversity across all metrics, with limited mutational variation and negative neutrality statistics (Table 8).

### 3.6. Phylogenetic Network Analysis

**Figure 6:**
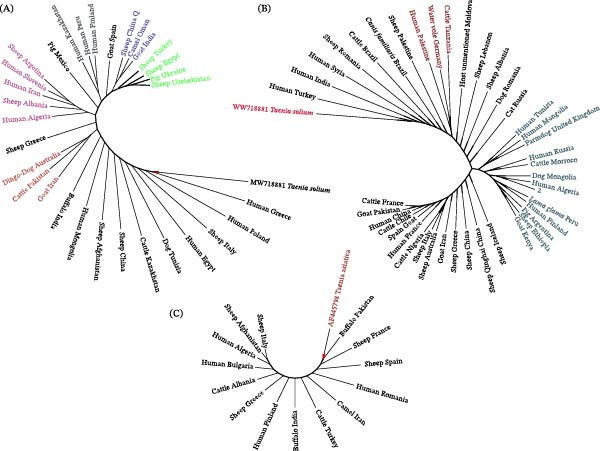
NJN phylogenetic network based on partial mitochondrial (A) *nad*1, (B) *cox*1 *sequences of E. granulosus* s.s. (G1), and (C) *nad*1 (G3) from global human and livestock hosts.

NJ Network (NJN) analysis based on mitochondrial *nad1* and *cox*1 sequences of G1 isolates and *nad*1 sequences of G3 isolates revealed a clear genetic separation between genotypes, while maintaining short intragenotype genetic distances (Figure 6). All G1 sequences clustered tightly, forming a dense network with limited branching, whereas G3 sequences formed a distinct and compact cluster.

Human and livestock derived isolates were interspersed throughout the G1 network, with no evidence of host‐specific clustering. The overall NJN topology corroborated the H network and diversity analyses, confirming weak phylogeographic structure and limited host‐associated genetic differentiation within *E. granulosus* s.s..

## 4. Discussion

This study provides the first comprehensive molecular characterization and global phylogeographic context of *E. granulosus* s.s. circulating in Afghanistan, integrating newly obtained mitochondrial sequence data with extensive publicly available datasets to elucidate genetic diversity, evolutionary relationships, and transmission dynamics.

The overwhelming predominance of the G1 genotype among Afghan isolates, with only a single G3 genotype, mirrors global trends wherein *E. granulosus* s.s. G1 is the most widely distributed and epidemiologically significant cause of CE in both humans and livestock (G1 accounts for the vast majority of human CE cases worldwide) as shown in large comparative mitochondrial analyses (whole mitogenome phylogeography) of over 200 globally distributed isolates [11].

Systematic reviews confirm that *E. granulosus* s.s. (G1–G3) is the dominant genotype across five continents and across diverse host species, especially in Asia, Europe, and Africa regions that were also well represented in our global dataset (Figures 1 and 2) [5]. This broad geographic presence highlights the capacity of G1 to maintain extensive transmission cycles in diverse socio‐ecological settings, consistent with its identification here in multiple host species without clear region‐specific segregation.

Our genetic diversity analyses revealed moderate to high Hd accompanied by low nucleotide diversity (π) across mitochondrial loci, a pattern frequently attributed to demographically expanding populations dominated by a few widely distributed Hs and many low frequency variants. Such signatures have been noted in other recent regional molecular studies of *E. granulosus* s.s., including in Iran, Pakistan, to mention few region, where high Hd and low π reflect prevalent G1 transmission and ongoing parasite dispersion [26]. The star‐like H networks (Figure 3, Supporting Information 2: Figure S2 and Supporting Information 3: Figure S3) reinforce this demographic interpretation, showing dominant central Hs shared across hosts and geographic regions with peripheral derived variants radiating outward, consistent with rapid and recent population expansion rather than long‐term isolation a pattern also reported in regional H surveys globally [27].

### 4.1. Neutrality and Sliding‐Window Analyses Support Genome‐Wide Homogeneity

Neutrality tests (Tajima’s *D* and Fu’s *F*_s_) yielded mostly insignificant departures from expectation for individual loci, while sliding‐window analyses showed consistently close‐to‐neutral *D* values and unimodal mismatch distributions for both *cox*1 and *nad*1 in G1 and G3 (Figure 5). These patterns are indicative of populations with limited demographic bottlenecks or selective sweeps affecting these loci, and are concordant with recent global mitochondrial surveys demonstrating neutral evolutionary dynamics in *E. granulosus* s.s. populations [27].

For G3, the particularly low nucleotide diversity and tightly clustered sliding‐window distribution suggest a narrow genetic base with limited mutational divergence, consistent with its documented lower global prevalence relative to G1 (e.g., in comparative mitogenome studies) [11].

### 4.2. Phylogenetic Relationships Affirm Genotype Integrity and Polyhost Transmission

Phylogenetic inference based on concatenated *cox*1–*nad*1 sequences clearly resolved *E. granulosus* s.s. into genotype‐specific clades, with all Afghan G1 isolates clustering with global G1 references and a distinct placement of the single Afghan G3 isolate (Figure 4). This clustering corroborates recent phylogeographic studies showing that G1 and G3 represent distinct mitochondrial lineages despite potential mito‐nuclear discordance identified in whole‐genome comparisons [28].

Notably, the absence of host‐specific clades in our G1 network, wherein isolates from sheep, cattle, and goats are interspersed, reflects extensive polyhost transmission cycles that have been observed in multiple regional studies, demonstrating that G1 maintains broad infectivity without strict host segregation [29].

### 4.3. Transcontinental H Sharing and Transmission Pathways

The global MJ networks for both *nad*1 and *cox*1 (Supporting Information 1: Figure S1, Supporting Information 2: Figure S2, Supporting Information 3: Figure S3) revealed intercontinental sharing of common Hs with only minor mutational divergence. Such patterns strongly indicate that geographic spread of *E. granulosus* s.s. lineages is driven by livestock movement, transhumance, and historical trade routes, rather than long‐term regional isolation. Similar conclusions emerged from a recent large‐scale mitogenomic phylogeography, which identified several strongly supported diffusion routes originating from historical livestock hubs and shaped by intensive animal trade [11].

This interpretation aligns with global reviews showing that Asia, Europe, and Africa have the highest overlap of overlapping *E. granulosus* s.s. occurrence, reflecting longstanding transcontinental connectivity of definitive hosts (canids) and intermediate hosts through human‐mediated livestock movements [5].

### 4.4. Implications for Epidemiology and Control Strategies

The combined genetic evidence underscores that *E. granulosus* s.s. populations are characterized by high connectivity and low regional differentiation, emphasizing that local control efforts must be integrated with regional animal movement regulations and One Health strategies to effectively reduce transmission. The lack of host‐specific structure further suggests that control targeting only one livestock species may be insufficient, and broader interventions encompassing dogs, livestock, and human communities are warranted a strategy strongly advocated in current One Health frameworks for CE control [5].

## 5. Limitations

Despite the strengths of our integrated analysis, several limitations remain. First, the use of mitochondrial markers, while valuable for phylogeography, provides a maternal lineage view and may underestimate recombination or nuclear gene flow; future studies incorporating nuclear loci or whole genomes would enhance resolution. Second, the single G3 isolate limits statistical power for comparative demographic analyses between G1 and G3 in Afghanistan. Third, the global comparative dataset, although comprehensive, is reliant on publicly available sequences, which are unevenly distributed geographically and may not fully capture unsampled diversity in under‐studied regions. Finally, restricted geographic sampling (Faryab Province only) and the absence of human clinical isolates from Afghanistan in this dataset constrains direct inference of zoonotic transmission pathways specific to Afghan populations. Nonetheless, our multilocus approach and integration of global data provide robust insights into the broad patterns of *E. granulosus* s.s. evolution and spread.

## 6. Conclusion

This study delivers the first integrated molecular and phylogeographic assessment of *E. granulosus* s.s. circulating in Faryab Province by extension Afghanistan within a global evolutionary context. Mitochondrial *cox1* and *nad1* analyses demonstrate a clear dominance of the G1 genotype in Afghan livestock, with only sporadic occurrence of G3, confirming the persistence of the classical dog livestock transmission cycle.

Despite regional sampling, Afghan isolates are genetically embedded within a globally connected *E. granulosus* s.s. population, as evidenced by star‐like H networks, extensive intercontinental H sharing, and minimal host‐ or geography‐specific structuring. The combination of moderate‐to‐high Hd and low nucleotide diversity indicates recent demographic expansion from a limited number of ancestral lineages rather than long‐term regional isolation.

Our findings highlight livestock movement and human‐mediated dispersal as dominant drivers of global parasite spread and underscore the limitations of geographically restricted control strategies. By placing Afghanistan within the global phylogeographic framework of *E. granulosus* s.s., this work provides critical baseline data for Central and South Asia and reinforces the need for coordinated One Health interventions targeting dog populations, livestock management, and transboundary animal movement.

**Nomenclature**
PBS: Phosphate‐buffered solution
PCR: Polymerase chain reaction
*cox*1: Cytochrome c oxidase subunit 1 gene
*nad*1: NADH dehydrogenase subunit 1 gene
CE: Cystic echinococcosis
MCMC: Markov chain Monte Carlo
Hd: Haplotype diversity
Fig. S: Figure supplementary.

## Author Contributions

Conceptualization: Tharheer Oluwashola Amuda, Sayed Ajmal Qurishi, Hong‐Bin Yan, and Li Li. Formal analysis: Tharheer Oluwashola Amuda and Sayed Ajmal Qurishi. Funding acquisition: Xue‐Nong Luo and Hong‐Bin Yan. Investigation: Tharheer Oluwashola Amuda, Sayed Ajmal Qurishi, Guo‐Dong Dai, and Wei‐Gang Chen. Methodology: Tharheer Oluwashola Amuda and Xue‐Nong Luo. Resources: Hong‐Bin Yan. Software: Tharheer Oluwashola Amuda and Guo‐Dong Dai. Supervision: Xue‐Nong Luo, Hong‐Bin Yan, Bao‐Quan Fu, and Wan‐Zhong Jia. Validation: Xue‐Nong Luo, Li‐Qun Wang, Hong‐Bin Yan, Sayed Ajmal Qurishi, and Mughees Aizaz Alvi. Visualization: Xue‐Nong Luo, Hong‐Bin Yan, and Awab Ghulam Rahim. Writing – original draft: Tharheer Oluwashola Amuda. Writing – review and editing: Xue‐Nong Luo, Li Li, Li Le, Yao‐Dong Wu, Mughees Aizaz Alvi, Nian‐Zhang Zhang, Wen‐Hui Li, Hong‐Bin Yan, Bao‐Quan Fu, Hong Yin, and Wan‐Zhong Jia.

## Funding

This work was financially supported by the Science and Technology Projects of Rikaze Autonomous Region, China (Grant RKZ2025ZD‐009), the Gansu Province Joint Research Fund (Grants 24JRRA807 and 25JRRA1164), the Innovation Program of Chinese Academy of Agricultural Sciences (Grants CAASTIP‐2026‐05 and CAAS‐ASTIP‐2021‐LVRI), the Agricultural Science and Technology Innovation Program (Grant CAAS‐ZDRW202607), the Gansu Provincial Research‐Industry Integration Science and Technology Empowerment Program (Grant 25FNNA002), the Major Science and Technology Project of Gansu Province (Grants 24ZD13NA008, 23ZDNA007, and 22ZD6NA001), and the NBCITS (Grant CARS‐37).

## Disclosure

All applications used in the analysis of data included in this manuscript has been clearly indicated in the method section. All authors have read this final manuscript and agreed for it to be published.

## Ethics Statement

This study was reviewed and approved by the Institutional Review Board of Lanzhou Veterinary Research Institute, Chinese Academy of Agricultural Sciences (Number LVRI‐IRB 201911).

## Consent

The authors have nothing to report.

## Conflicts of Interest

The authors declare no conflicts of interest.

## Supporting Information

Additional supporting information can be found online in the Supporting Information section.

## Supporting information


**Supporting Information 1** Figure S1. Median Joining (MJ) network of nad1 mitochondrial gene sequences from 13 E. granulossus s.s. (G3) worldwide.

**Supporting Information 2** Figure S2. Median Joining (MJ) network of nad1 gene (894 bp) mitochondrial gene sequences of E. granulossus s.s. (G1) worldwide.

**Supporting Information 3** Figure S3. Median Joining (MJ) network of cox1 gene (1876 bp) mitochondrial gene sequences of E. granulossus s.s. (G1) worldwide.

**Supporting Information 4** Figure S4. Global distribution of E. granulossus s.s. (G1) worldwide haplotypes based on the mitochondrial cox1 1874 bp.

**Supporting Information 5** Graphical abstract.

## Data Availability

All data generated or analyzed in this paper are provided in this paper and the data that support the findings of this study are openly available in GenBank at https://www.ncbi.nlm.nih.gov/genbank/, Accession Number for G1 genotype (PQ305597–PQ305606 and PQ821697–PQ821702) and G3 genotype (PV610729).
