# Supplementary figures and images for: Global Comparative Phylogeography of E. granulosus s.s. Inferred From Mitochondrial DNA: Integrated Datasets of GenBank and Newly Characterized Isolates From Afghanistan
Source: Transbound Emerg Dis. 2026 Jul 16;2026:2401137. doi: 10.1155/tbed/2401137 (PMC13373698; doi:10.1155/tbed/2401137)

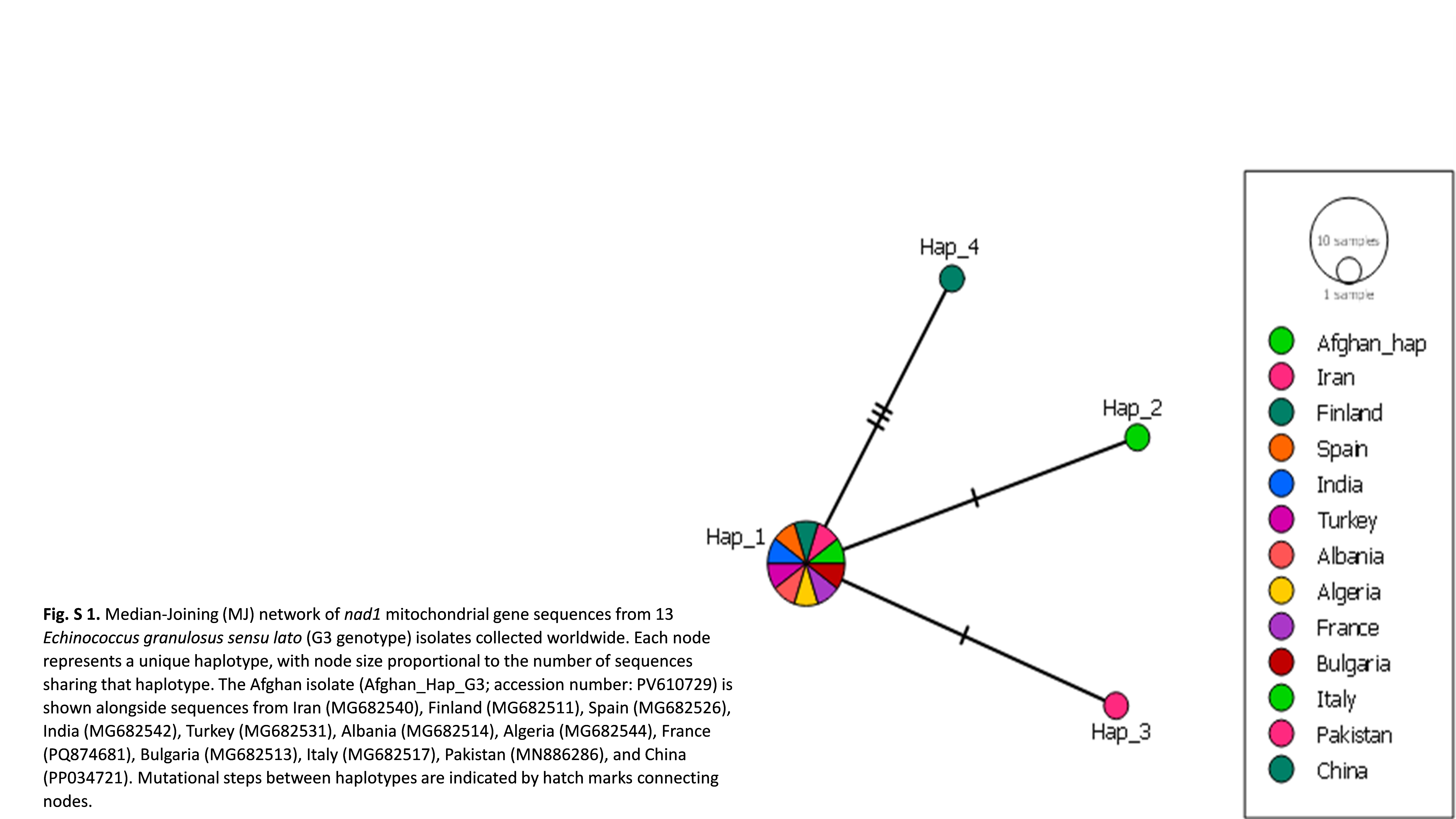

Supplement: Supplementary file 1 — Supporting Information 1 Figure S1. Median Joining (MJ) network of nad1 mitochondrial gene sequences from 13 E. granulossus s.s. (G3) worldwide. [file TBED-2026-2401137-s005.jpg]

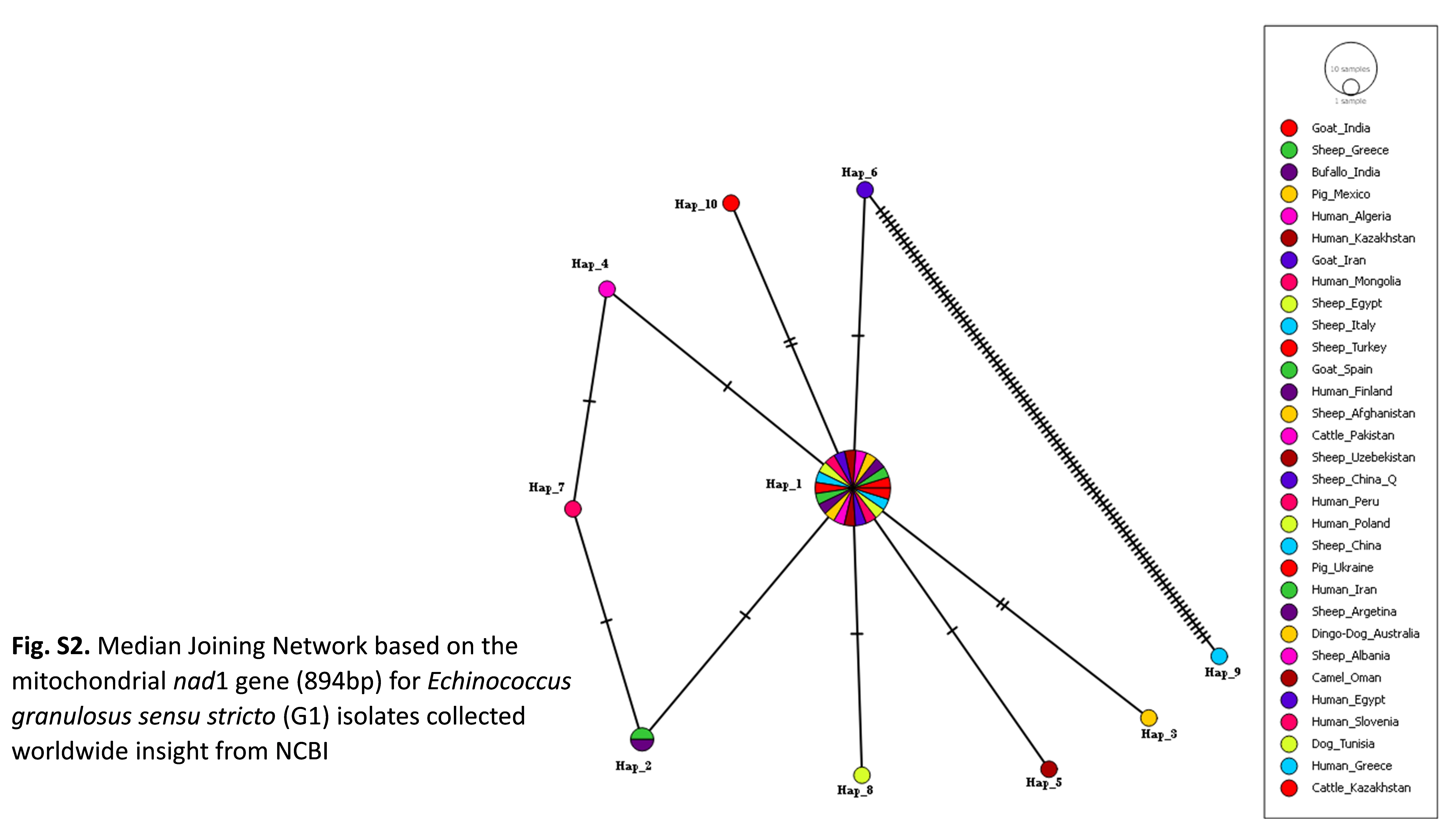

Supplement: Supplementary file 2 — Supporting Information 2 Figure S2. Median Joining (MJ) network of nad1 gene (894 bp) mitochondrial gene sequences of E. granulossus s.s. (G1) worldwide. [file TBED-2026-2401137-s004.jpg]

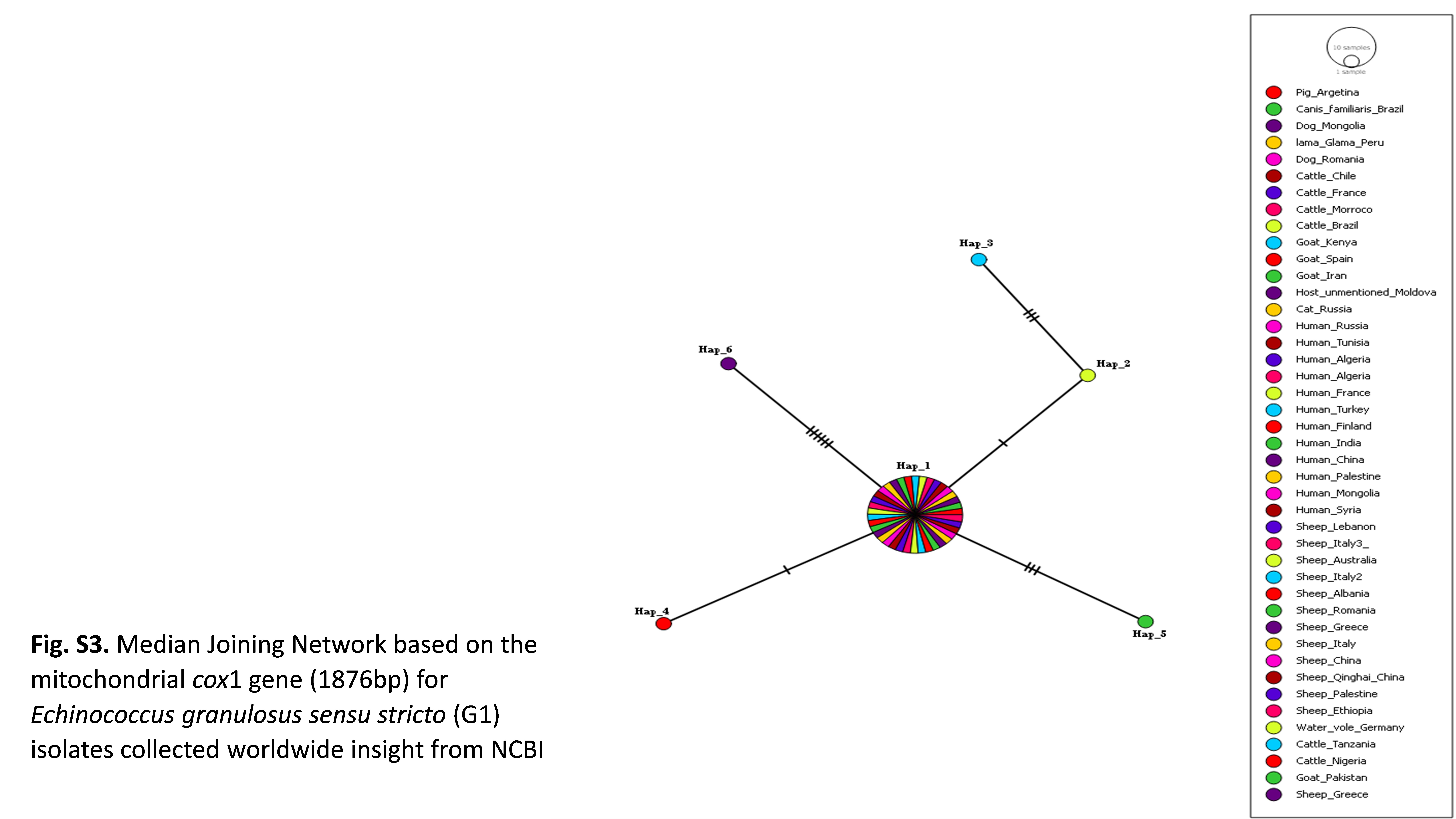

Supplement: Supplementary file 3 — Supporting Information 3 Figure S3. Median Joining (MJ) network of cox1 gene (1876 bp) mitochondrial gene sequences of E. granulossus s.s. (G1) worldwide. [file TBED-2026-2401137-s003.jpg]

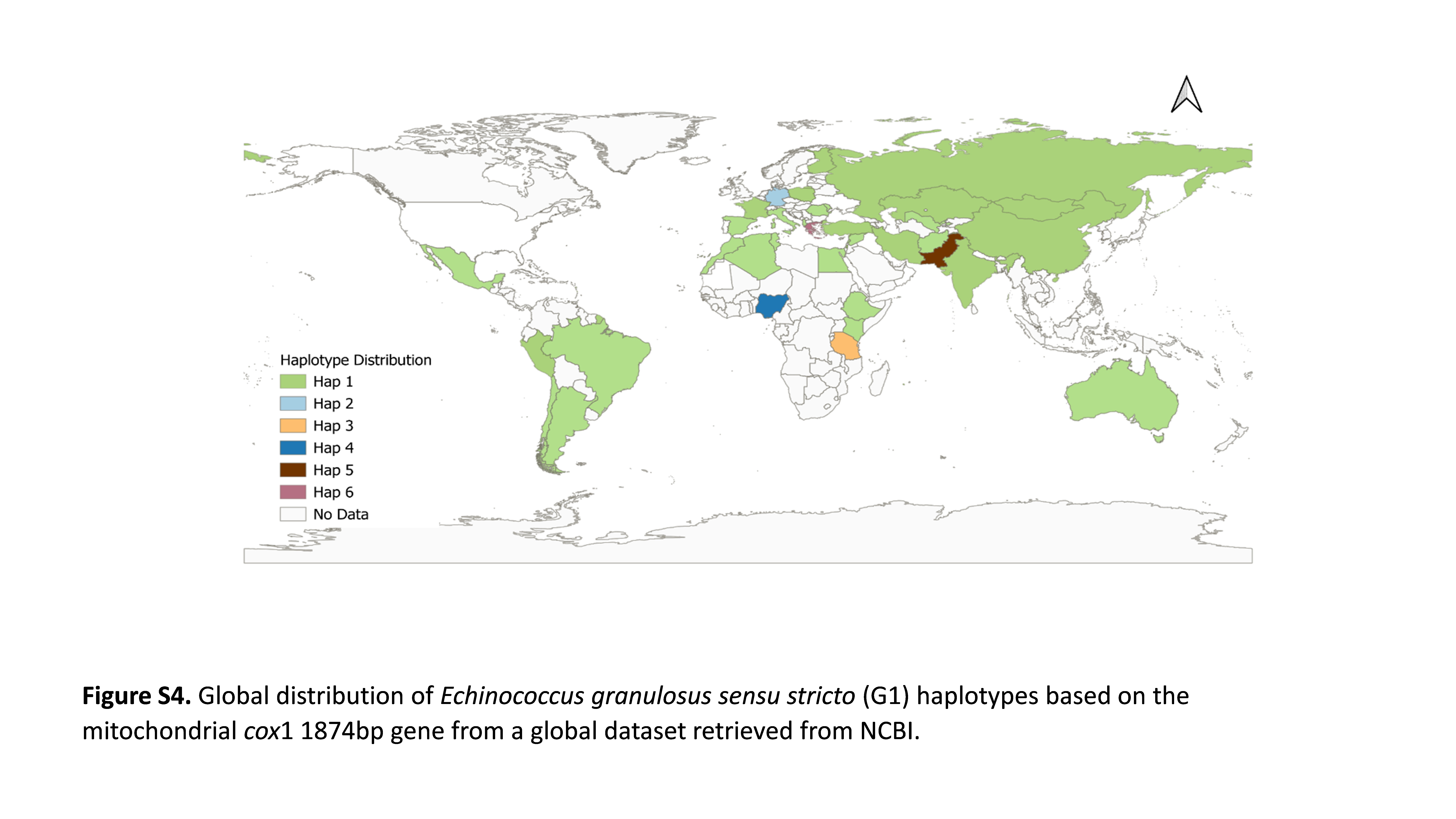

Supplement: Supplementary file 4 — Supporting Information 4 Figure S4. Global distribution of E. granulossus s.s. (G1) worldwide haplotypes based on the mitochondrial cox1 1874 bp. [file TBED-2026-2401137-s002.jpg]

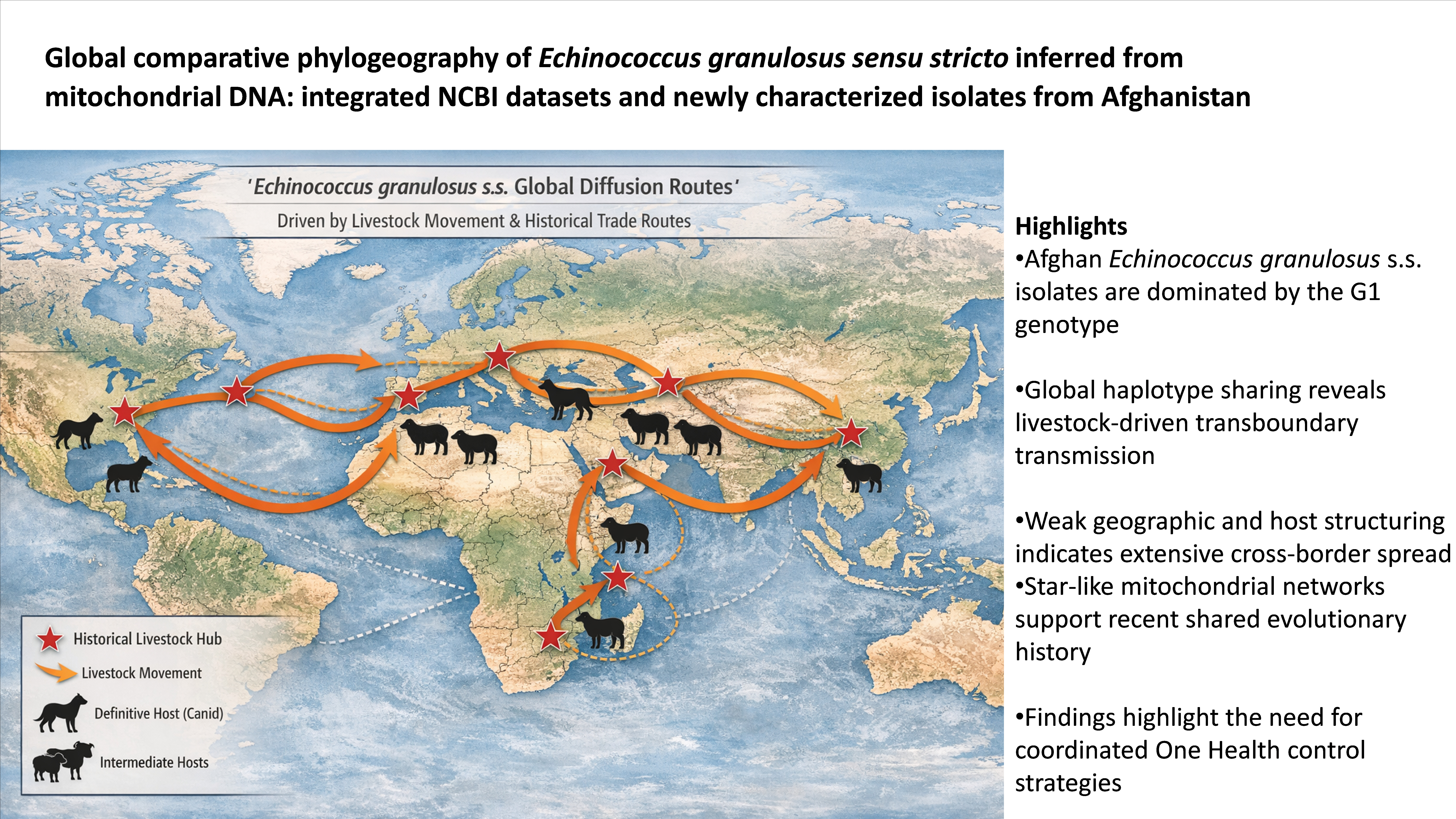

Supplement: Supplementary file 5 — Supporting Information 5 Graphical abstract. [file TBED-2026-2401137-s001.jpg]
